# Successful treatment of atypical cesarean scar defect using endoscopic surgery

**DOI:** 10.1186/s12884-015-0730-x

**Published:** 2015-12-22

**Authors:** Hirotaka Masuda, Hiroshi Uchida, Tetsuo Maruyama, Kenji Sato, Suguru Sato, Mamoru Tanaka

**Affiliations:** Department of Obstetrics and Gynecology, Keio University School of Medicine, 35 Shinanomachi, Shinjuku-ku, Tokyo, 160-8582 Japan

**Keywords:** Cesarean scar syndrome, Isthmocele, Postmenstrual bleeding, Infertility, Laparoscopic surgery, Hysteroscopic surgery

## Abstract

**Background:**

Cesarean scar syndrome results from a postoperative defect of the uterine isthmus, also known as an isthmocele. Patients present with gynecological symptoms, such as abnormal genital bleeding or infertility, after cesarean delivery. Although the cesarean rate is increasing worldwide, this syndrome is not widely known.

**Case presentation:**

A 43-year-old G2P1 Japanese woman with atypical cesarean scar syndrome had a 3-year history of secondary infertility and postmenstrual brown discharge. Laparoscopic and hysteroscopic exploration revealed a cesarean scar defect connected to a small cavity in the myometrium: this was not an endometrial cavity or a uterine diverticulum. After endoscopic excision of the cavity, the brown discharge resolved, and the patient achieved ongoing pregnancy on her third attempt at intrauterine insemination.

**Conclusion:**

Consensus is still lacking regarding the diagnosis and treatment of cesarean scar defect. However, the gynecologists should be aware that cesarean scar syndrome can have scar defects forming cavities of unusual shapes and features. Surgical correction of these defects will often improve postmenstrual bleeding and subfertility in these cases.

## Background

Scar defects at the isthmus uteri, also known as cesarean scar defects or isthmoceles, are often found after cesarean delivery [1]. Cesarean scar defects are being more commonly reported but the incidence varied between 24 and 84 % [2]. Some women are asymptomatic, but others may have gynecologic symptoms [2] such as postmenstrual spotting, prolonged menstruation, continuous brown discharge, chronic pelvic pain [3], and secondary infertility [4, 5]. These symptoms, taken together, have been closely investigated and are called cesarean scar syndrome [1]. Other problems associated with cesarean scar defect are a higher risk of complications during subsequent pregnancy [6], such as dehiscence, placenta previa or accreta [7] and cesarean scar ectopic pregnancy [8], and difficulty with gynecologic procedures like uterine evacuation, hysteroscopy, and intrauterine-device insertion [9].

Recently, the clinical relevance of cesarean scar defects has attracted an increasing amount of attention, with more review articles published, because cesarean rates are rising worldwide [2, 9–12]. While symptom relief has been reported after surgical intervention, the etiology of the syndrome itself and therapeutic effects on the risk of subsequent pregnancy complications or the difficulty of future gynecologic procedures are still unclear. Additionally, a worldwide, unified detection method, diagnostic criteria, and treatment protocol are currently not available. Therefore, a standardized definition for cesarean scar defect and thorough long-term follow-up are urgently required.

We encountered a rare case of an atypical cesarean scar defect connected to a small cavity in the myometrium. To the best of our knowledge, this presentation has not yet been reported. We report our successful treatment of this case.

## Case presentation

**Fig. 1:**
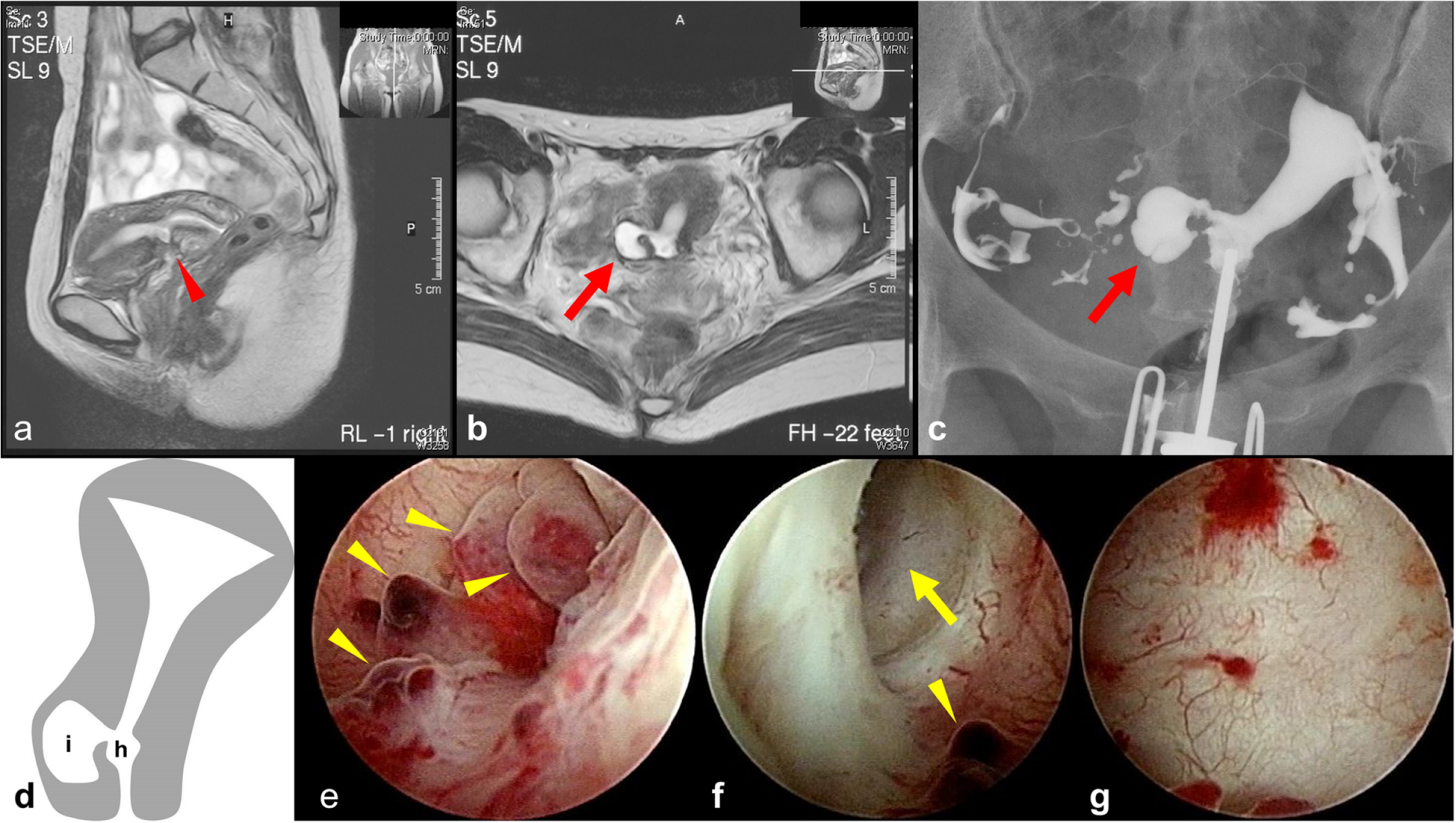
**a** T2-weighted magnetic resonance imaging (MRI): red arrowhead indicates the cesarean scar defect. **b** T2-weighted MRI: the appearance of the cavity (*red arrow*) is suspicious for a bicornuate uterus. **c** Hysterosalpingography: the right fallopian tube is not connected to the small cavity (*red arrow*), ruling out a bicornuate uterus. **d** Schematic of the patient's uterus. (a: The cesarean scar defect, b: The small cavity) (**e**-**g**) Hysteroscopic images. **e** Polyps (*yellow arrowheads*) around the cesarean scar defect (corresponding to “a” on schematic). **f** The entrance (*yellow arrow*) of a small cavity viewed from the cesarean scar defect (corresponding to “h” on schematic). **g** The inside wall of the small cavity (corresponding to “i” on schematic). The wall of the cavity is smooth and hard, with no endometrial tissue present

A 43-year-old Japanese woman, gravida 2 para 1, had a 3-year history of secondary infertility but had easily conceived her first child, 6 years ago, 3 months after discontinuing contraception. Her menstrual cycle was regular at 28–30 days, with menses lasting for 5 days, but since her first child she experienced 2 weeks each month of postmenstrual brown discharge. Magnetic resonance imaging (MRI) was performed (Fig. 1a, b), raising the suspicion of a bicornuate uterus, but this was not borne out on hysterosalpingography (Fig. 1c). Hysteroscopy revealed polyps at the site of the defect (Fig. 1e) and a cavity bulging out to the patient’s right from the cesarean scar defect (Fig. 1d, f, g). The cavity in question was larger than the cesarean scar defect, but less than half the size of the endometrial cavity. We speculated that a uterine diverticular hernia had arisen intraperitoneally from the myometrial incision site and thus planned a laparoscopic excision of the diverticulum and repair of the cesarean scar defect.

**Fig. 2:**
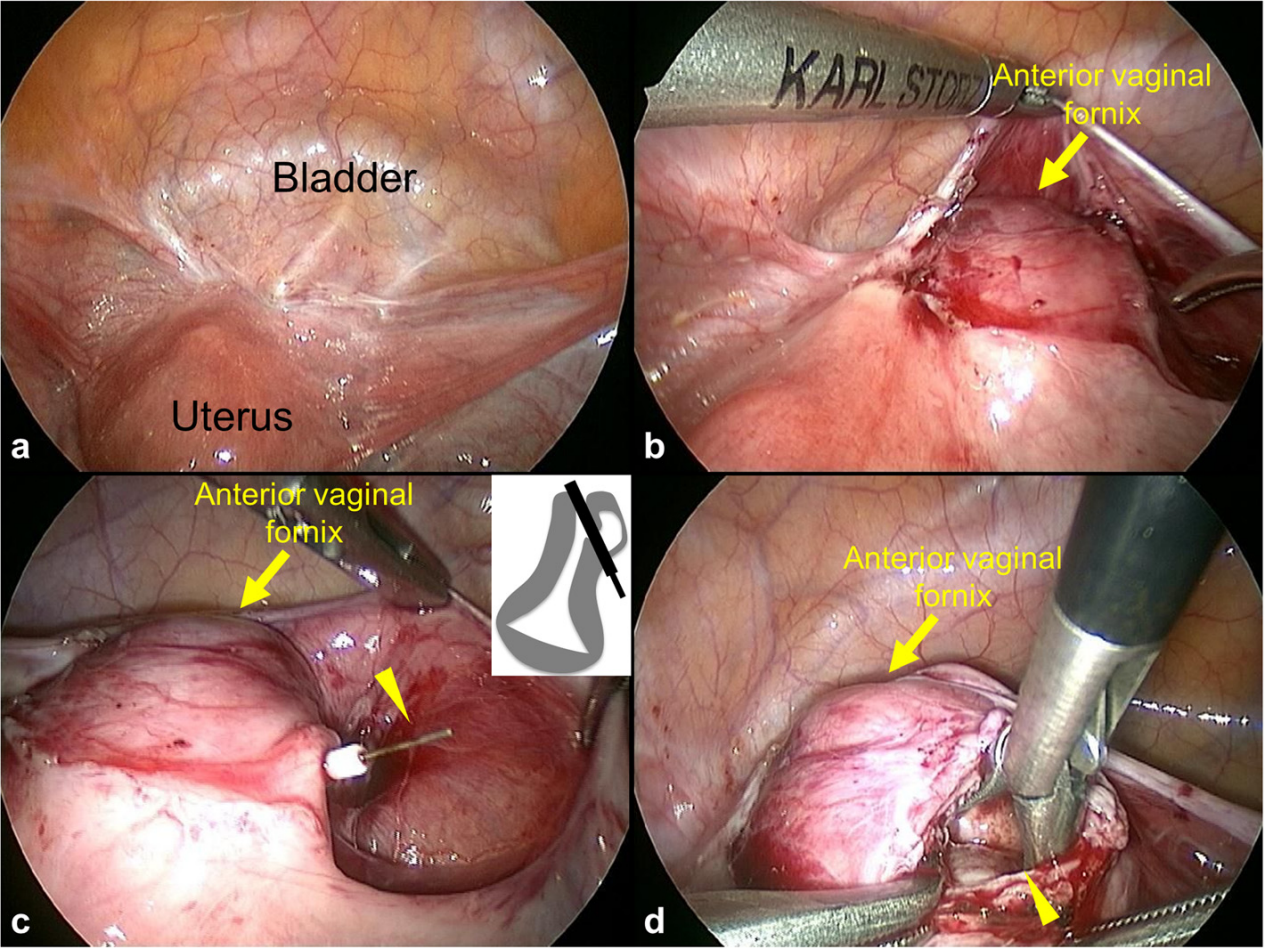
Laparoscopic images. **a** No extrauterine cystic mass is present. **b** No uterine diverciculum is detected under the vesicouterine pouch. The yellow arrow indicates the anterior vaginal fornix. **c** The bipolar device (*yellow arrowhead*) penetrating through the myometrium. Inset: schematic illustration. **d** The opened cavity (*yellow arrowhead*)

Surprisingly, laparoscopic intraperitoneal exploration did not reveal a uterine diverticulum (Fig. 2a). The cavity was not palpable even under the vesicouterine pouch (Fig. 2b). Therefore, we introduced a hysteroscopic bipolar device through the cavity to perforate the myometrium (Fig. 2c). We then cut into the myometrium, along the device, to open the cavity (Fig. 2d). After the cavity walls were resected and debrided, the cavity space was closed with absorbable sutures under laparoscopic guidance. We confirmed the obliteration of the cavity using a hysteroscope, then removed polyps and scar tissues at the cesarean scar defect hysteroscopically. A leak test was performed using indigo carmine solution before completion of surgery. Pathological examination revealed that the polyps at the cesarean scar defect site (Fig. 1e) were composed of endometrial glands and the cavity wall (Fig. 1g) consisted of hyalinized myometrium.

The patient’s postmenstrual discharge stopped immediately after the surgery. A hysteroscopic examination was performed 3 months later and confirmed that the cavity had not recurred; the patient was cleared to attempt pregnancy. She conceived a month later with first attempt at intrauterine insemination. Unfortunately, this pregnancy ended in a miscarriage at 6 weeks' gestation. However, she conceived again through her third intrauterine insemination and, in consideration for the vulnerability of the repair site, cesarean section was performed at 37 weeks of pregnancy.

We were able to successfully treat a patient with atypical cesarean scar defect. In this case, the cesarean scar defect was connected to a cavity that bulged into the myometrium, a finding that has not yet been reported. The patient’s postmenstrual discharge resolved after surgery, and her infertility has also resolved, as she was able to get pregnant twice out of three attempts of intrauterine insemination in spite of 3 years of infertility prior to our intervention.

Hyalinized myometrium of the cavity wall (Fig. 1g) indicates an old myometrial scar; this differs from a true uterine diverticulum that is lined by glandular tissue [13, 14]. Our patient’s findings also differed from those of a secondary uterine diverticulum, which is an extrauterine swelling of a severe cesarean scar defect [13, 14]. In our patient, the sacculation of hyalinized myometrium in the right uterine sidewall encourages us to imagine that unsutured lacerations at the right edge of the myometrial incision gave rise to the small cavity. Traditionally, the surgeon stands on the patient’s right and the right inner edge of the myometrial incision could be a blind spot; this point has never been discussed before. Gubbini et al. analyzed 26 patients with cesarean scar defect and reported that the defects appeared anteriorly, near the right side [15]. They usually stand on the patient’s right as well (personal communication), which supports our speculation. Therefore, we propose that extra attention should be paid to reduce interspaces when suturing the inner edge of the cesarean incision at uterine closure. Furthermore, several studies have discussed closure technique of uterine incision as a risk for cesarean scar defect, although this remains controversial [11, 16–21]. Taken together, it may be concluded that double-layer uterine closure, especially with endometrial suturing, is the best way to prevent scar defects. The incidence of cesarean scar defect increases with multiple cesarean sections but there may other potential risk factors [22]. However, of all possible risks/predisposing factors, only uterine incision closure technique can be controlled for the primary prevention, which is undoubtedly important.

Regarding her postmenstrual brown discharge, reduced contractility in the lower uterine segment might slow the drainage of menstrual blood [23], or the blood could accumulate in the cavity with slow subsequent drainage [21]. Another potential source of bleeding is the polyps (Fig. 1e), which are found in 16 % of cesarean scar syndrome patients [1]. Endometrial abnormalities around the defect could cause abnormal bleeding, such as overhanging congested endometrium, fragmentation and breakdown of the endometrium [1], and inclusion of endometrial tissue within the scar [24]. Endometrial suturing at the time of uterine closure could bring endometrium into the correct position and reduce endometrial abnormalities and abnormal bleeding, which may be an important suturing technique as mentioned above. In any event, the slow drainage of menstrual blood must negatively affect sperm viability, sperm swim-up, and embryo implantation [2]. In our patient, obliteration of the cavity and removal of the polyp were thought to improve both her discharge and infertility. Therefore, even in situations e.g. in developing area where endoscopic (minimally invasive) surgical facilities are not available, open surgery remains a reasonable option.

Although, retrospectively, the MRI and hysterosalpingography images of this case were different from a common cesarean scar defect, the correct diagnosis was, indeed, quite difficult before surgery despite the use of transvaginal ultrasound, MRI, hysterosalpingography, and hysteroscopy. Sonohysterography might have helped to make a more accurate diagnosis [25]. Finally, both laparoscopy and hysteroscopy were required to identify all aspects of this irregular defect. Regarding surgical treatment options of cesarean scar syndrome, operative hysteroscopy, conventional or robotic-assisted laparoscopic surgery and vaginal surgery have been reported [12, 26–28]. However, we needed both laparoscopic and hysteroscopic surgery to complete the repair. Clearly, a consistent methodology of diagnosis and treatment, taking into consideration such atypical cases, is needed.

## Conclusions

In this report, we propose a potential treatment for atypical cesarean scar defect. As the cesarean rate is increasing worldwide, the possibility of encountering a patient with an atypical cesarean scar defect is rising. The gynecologists should be aware that cesarean scar syndrome can present with scar defects of atypical shapes or features. Thus the diagnostic evaluation and therapeutic options should be carefully evaluated.

### Consent

Written informed consent was obtained from the patient for publication of this Case report and any accompanying images. A copy of the written consent is available for review by the Editor of this journal.

## Abbreviation

MRI: Magnetic resonance imaging
